# Case Report: Evolution of intratumoral spatial heterogeneity assessed by [^68^Ga]Ga-DOTA-NOC PET/CT during dinutuximab beta therapy in pediatric high-risk neuroblastoma

**DOI:** 10.3389/fped.2026.1829673

**Published:** 2026-07-21

**Authors:** Yueran Chen, Qinfeng Xu, Haoyan Zhang, Yongjun Fang, Guoqiang Shao

**Affiliations:** 1 Department of Nuclear Medicine, Nanjing First Hospital, Nanjing Medical University, Nanjing, China; 2 Department of Nuclear Medicine, Jiangsu Province Hospital of Chinese Medicine, The Affiliated Hospital of Nanjing University of Chinese Medicine, Nanjing, China; 3 School of Basic Medicine and Clinical Pharmacy, China Pharmaceutical University, Nanjing, China; 4 Department of Hematology, Children's Hospital of Nanjing Medical University, Nanjing, China; 5 Department of Nuclear Medicine, Maanshan People's Hospital, Maanshan, China

**Keywords:** [^68^Ga]Ga-DOTA-NOC PET/CT, dinutuximab beta, high-risk neuroblastoma, intratumoral heterogeneity, molecular imaging

## Abstract

Neuroblastoma, an embryonal neuroendocrine tumor, exhibits substantial biological heterogeneity and frequently expresses somatostatin receptors (SSTRs). Consequently, [^68^Ga]Ga-DOTA-NOC PET/CT is utilized as a targeted functional imaging modality to evaluate SSTR expression, though its utility in monitoring responses to multimodal immunotherapy remains complex. We report a 4-year-old male with high-risk neuroblastoma (Stage M) evaluated via serial [^68^Ga]Ga-DOTA-NOC PET/CT. Baseline imaging revealed a retroperitoneal tumor [maximum standardized uptake value (SUVmax) 6.5] and skeletal metastases. Following four cycles of induction chemotherapy combined with dinutuximab beta, a post-therapeutic PET/CT performed prior to surgical resection revealed that the primary mass structurally regressed and skeletal lesions resolved. However, a focal region within the residual primary tumor exhibited a discordant radiotracer uptake increase (SUVmax rising from 3.1 to 17.4). Histopathological evaluation confirmed this hyper-avid region as a viable, poorly differentiated neuroblastoma subclone. Consequently, while [^68^Ga]Ga-DOTA-NOC PET/CT assists in localizing viable residual disease and guiding surgical navigation, elevated SSTR accumulation following immunotherapy necessitates cautious multidisciplinary evaluation.

## 1 Introduction

Neuroblastoma is one of the most common extracranial solid neoplasms in childhood, representing a form of embryonal neuroendocrine tumor (1, 2). High-risk neuroblastoma exhibits substantial intratumoral heterogeneity, contributing to disease relapse and treatment resistance (3, 4). Anti-GD2 monoclonal antibodies, such as dinutuximab beta, are integrated into induction and consolidation regimens (5, 6). Monitoring therapeutic responses in these patients relies on molecular imaging. While [^123^I]mIBG remains the conventional standard, [^68^Ga]Ga-DOTA-NOC PET/CT is utilized to evaluate somatostatin receptor (SSTR) expression (7, 8). Recently, the published Appropriate Use Criteria (AUC) have further standardized the application of SSTR PET imaging in neuroblastoma, underscoring its pivotal role in specific clinical and therapeutic scenarios (9). Conventionally, increased SSTR accumulation within residual tumor tissue suggests therapy-induced benign neuronal maturation (7, 10). However, the selective pressure from immunotherapy on the tumor microenvironment may alter receptor expression patterns, and the spatial evolution of SSTR under these conditions requires further investigation. This report details a case wherein focal SSTR upregulation following dinutuximab beta therapy corresponded to a viable, therapy-resistant subclone rather than benign differentiation. These findings demonstrate the application of receptor-targeted molecular imaging in assessing spatial heterogeneity. Concurrently, they suggest that elevated radiotracer accumulation following immunotherapy may represent distinct biological entities, indicating a requirement for multidisciplinary evaluation in response assessment.

## 2 Case description and diagnostic assessment

**Figure 1:**
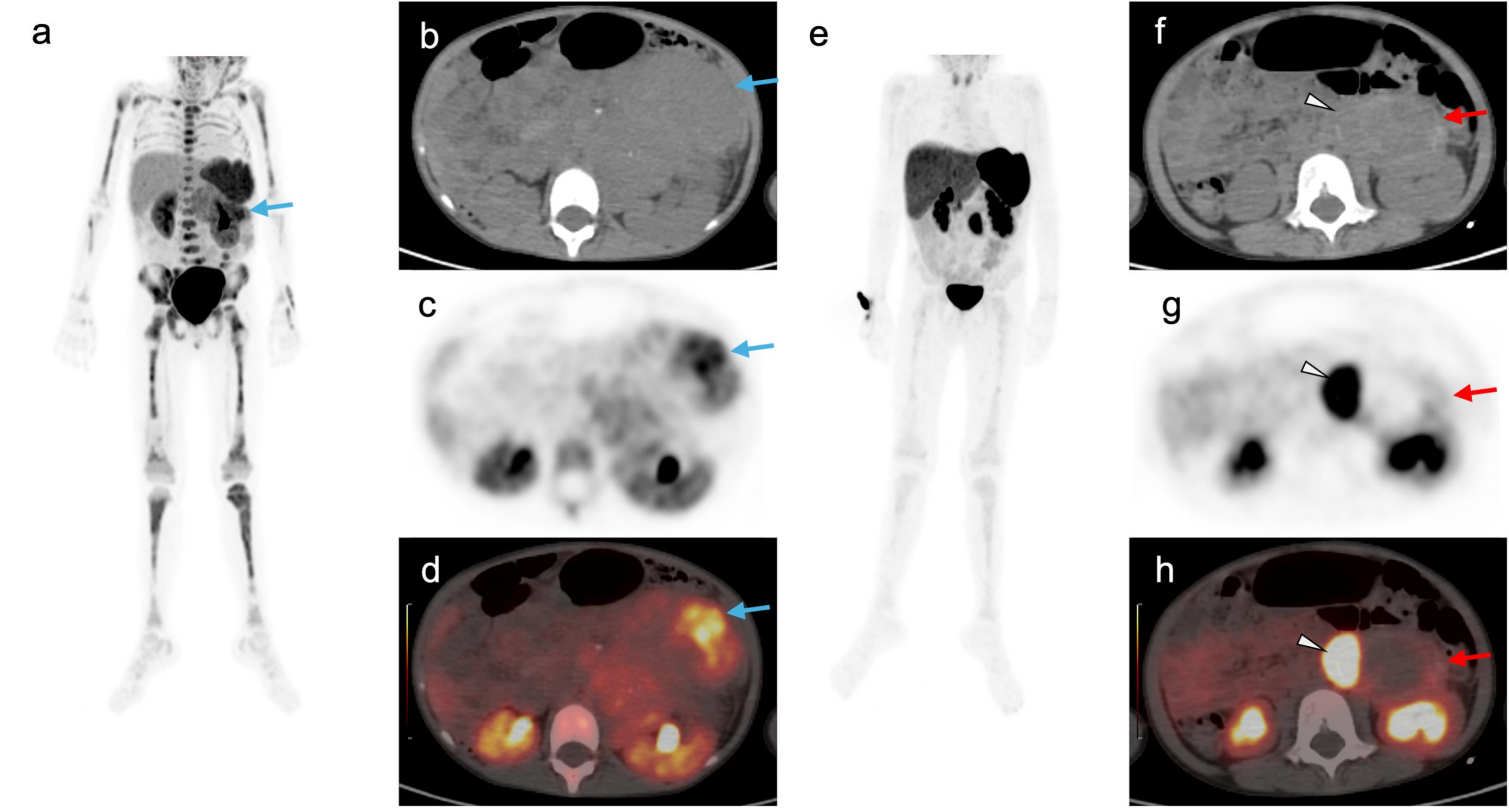
**(a)** maximum intensity projection (MIP); **(b)** axial CT; **(c)** axial PET; **(d)** fused PET/CT; **(e)** MIP; **(f)** axial CT; **(g)** axial PET; and **(h)** fused PET/CT.

A 4-year-old male presented with an abdominal mass and elevated serum neuron-specific enolase (NSE). Baseline [^68^Ga]Ga-DOTA-NOC PET/CT identified a left retroperitoneal primary tumor (12.7 × 7.9 × 14.6 cm; SUVmax 6.5; Figures 1a–d, blue arrow) and extensive skeletal metastases exhibiting radiotracer accumulation (Figure 1a). The diagnosis was INRG stage M high-risk neuroblastoma.

The patient received four cycles of preoperative induction chemotherapy combined with dinutuximab beta (20 mg/vial). The total dose of immunotherapy was 40–60 mg per cycle (adjusted by body surface area), administered via 24-hour continuous intravenous infusion for 10 consecutive days per cycle. Post-induction [^68^Ga]Ga-DOTA-NOC PET/CT, performed following the completion of four induction cycles, demonstrated primary tumor dimension reduction (7.0 × 3.9 × 6.8 cm; Figures 1e–h, red arrow) and resolution of radiotracer uptake in the skeletal lesions. A discrete focal area within the residual primary mass exhibited a discordant increase in radiotracer accumulation, with the SUVmax rising from 3.1 to 17.4 (Figures 1e–h, white arrowhead).

To achieve local control, the localized radiotracer accumulation identified via [^68^Ga]Ga-DOTA-NOC PET/CT guided surgical navigation for the targeted resection of the residual mass. Macroscopic evaluation of the resected specimen revealed necrosis (approximately 50%), focal calcification, and fibroplasia (10%). Crucially, these macroscopic areas of necrosis spatially corresponded to the non-avid portions of the mass observed on the PET/CT images. Microscopic evaluation of the high-uptake focal area identified a viable tumor bed containing poorly differentiated neuroblastoma (20%) and differentiating nodular ganglioneuroblastoma components (20%). Immunohistochemical profiling of this specific region demonstrated a Ki67 proliferation index of 12%+ alongside confirmed SSTR2 and SSTR5 positivity. Following surgical excision, the patient completed standardized post-operative consolidation therapy. At the most recent follow-up (approximately 8 months post-surgery), clinical evaluation demonstrated no evidence of disease (NED) or relapse. Routine monitoring continues in accordance with established clinical guidelines.

### 2.1 Timeline

**Figure 2:**
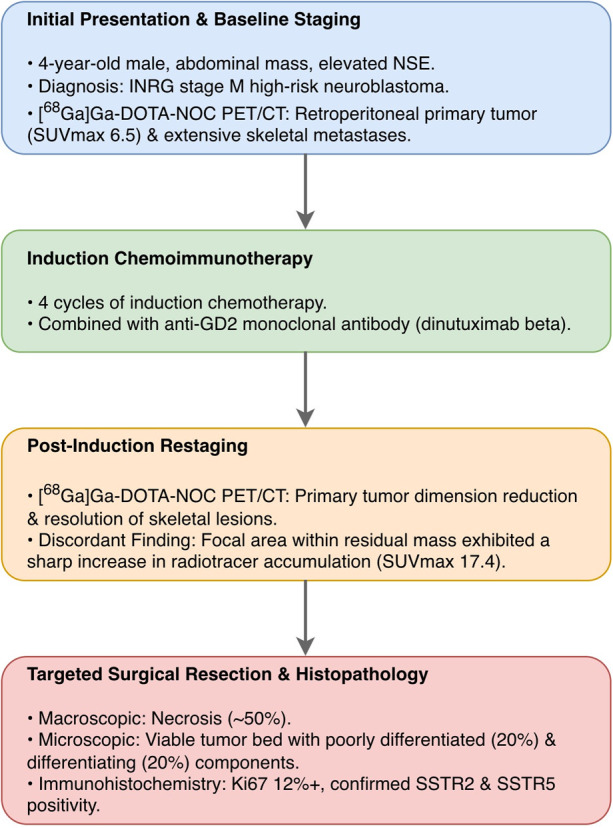
Clinical timeline outlining the sequence of diagnostic imaging, dinutuximab beta-based induction therapy, and subsequent targeted surgical intervention.

The clinical timeline detailing the sequence of diagnostic evaluations, induction therapy, and surgical intervention is illustrated in Figure 2.

## 3 Discussion

Evaluating therapeutic response following dinutuximab beta therapy revealed molecular-morphological discordance. Histopathological analysis of the resected specimen provided the biological basis for this finding. The coexistence of poorly differentiated (20%) and differentiating neuroblastoma components (20%), alongside elevated proliferative activity (Ki67 index 12%+), within the high-uptake focus suggests spatial heterogeneity. Immunohistochemical confirmation of SSTR2 and SSTR5 expression corresponds to the receptor affinity profile of [^68^Ga]Ga-DOTA-NOC. Compared to the therapy-induced necrosis (approximately 50%) in surrounding tissues, this molecular composition suggests an adaptive clonal selection of a therapy-resistant subpopulation driven by treatment pressure (4, 11, 12).

Interpreting persistent or elevated SSTR positivity during immunotherapy requires contextual analysis. Conventionally, SSTR2 overexpression correlates with neuronal differentiation and favorable prognostic features (7, 8), leading to the assumption that localized uptake increases represent maturing cellular clones. In this post-induction setting, focal SSTR upregulation (SUVmax rising from 3.1 to 17.4) corresponded to a viable malignant subclone. This finding may reflect an adaptive evolutionary response and receptor upregulation induced by the therapeutic stress of dinutuximab beta and chemotherapy (5, 13, 14). Elevated SSTR accumulation may therefore reflect a spectrum of biological responses, potentially indicating either benign differentiation or the spatial persistence of viable malignancy.

These observations demonstrate the application of [^68^Ga]Ga-DOTA-NOC PET/CT in evaluating resistant disease foci that evade conventional morphological assessment (15, 16). Furthermore, a recent meta-analysis indicated that SSTR PET surpasses most other molecular imaging modalities, such as FDG and MIBG, in the comprehensive evaluation of primary and metastatic neuroblastoma (17). The detection of discordant radiotracer accumulation, characterized by increased uptake within a structurally regressing primary lesion, guided surgical navigation for the targeted resection of the viable tumor bed. Integrating these receptor expression dynamics into clinical evaluations could assist in defining the extent of viable residual disease, thereby optimizing multidisciplinary local control strategies in pediatric high-risk neuroblastoma.

In conclusion, this case highlights the noteworthy utility of [68 Ga]Ga-DOTA-NOC PET/CT in localizing viable residual neuroblastoma and assessing spatiotemporal tumor heterogeneity following immunotherapy. While elevated SSTR expression conventionally implies benign maturation, it may also indicate a therapy-resistant subclone, underscoring the necessity of cautious multidisciplinary evaluation and imaging-guided surgical intervention.

## 4 Patient Perspective

The patient’s legal guardians expressed understanding regarding the high-risk nature of the diagnosis. They acknowledged the utility of advanced functional imaging in identifying the covert, persistent disease focus, which directly guided the subsequent surgical resection and consolidated the overall treatment strategy.

## Data Availability

The original contributions presented in the study are included in the article. Further inquiries can be directed to the corresponding author.
